# GPs’ perspectives of the patient encounter – in the context of standardized cancer patient pathways

**DOI:** 10.1080/02813432.2020.1753388

**Published:** 2020-04-21

**Authors:** Cecilia Hultstrand, Anna-Britt Coe, Mikael Lilja, Senada Hajdarevic

**Affiliations:** a Department of Nursing, Umeå University, Umeå, Sweden;; b Department of Public Health and Clinical Medicine, Family Medicine, Umeå University, Umeå, Sweden;; c Department of Sociology, Umeå University, Umeå, Sweden;; d Department of Public Health and Clinical Medicine, Unit of Research, Education, and Development, Östersund Hospital, Umeå University, Umeå, Sweden

**Keywords:** Encounter, interpretation, symptoms, primary care, cancer patient pathways, general practitioners, symbolic interactionism

## Abstract

**Objective:** We aim to explore how GPs assign meanings and act upon patients’ symptoms in primary care encounters in the context of standardized cancer patient pathways (CPPs).

**Design, setting and subjects:** Thirteen individual interviews were conducted with GPs, at primary healthcare centers (*n* = 4) in one county in northern Sweden. Interviews were analyzed using grounded theory method. The results were then linked to symbolic interactionism.

**Main outcome measures:** GPs’ perspectives about assigning meanings to patients’ presented symptoms and perception about CPPs.

**Results:** In the encounter, GPs engaged in two simultaneous interactions, one with patients’ symptoms – and the other with CPPs. The core category *Disentangling patients’ care trajectory* consists of three categories, interpreted as GPs’ strategies developed to assign meaning to symptoms. These strategies are carried out not in a straightforward manner but rather in a conflicting way, illuminating the complexity of GPs’ daily work.

**Conclusions:** Interacting with patients is vital for assigning meaning to presented symptoms. However, nowadays GPs are not only required to interact with patients, they are also required to interact with CPPs. These standardized routines might create pressure and demands on GPs, especially for those experiencing a lack of information about CPPs. Beside of carrying out the challenging patient/person-centered dialogues and interpreting presented symptoms, GPs also need to link the interpreted symptoms to CPPs. Therefore, it is essential that GPs are given opportunities at their workplaces to continuously be informed and be supported in order to practice CPPs and thereby optimize trajectories for patients undergoing cancer diagnostics.

**Key points**
 Current awareness:
 • GPs deliberation about patients’ trajectories is a complex process, often dealing with vague symptoms. How CPPs influence this process within the encounter has not been studied.
 Main statements:  • GPs in our study were involved in two simultaneous interactions, one with patients’ symptoms in the encounter – and the other with CPPs within the healthcare organization.
 • Symbolic interactionism helped capture how GPs deliberated about conflicting and paradoxical aspects of the encounter, in terms of balancing two contradictory ways of action that GPs face when providing patient/person-centered care and linking to CPPs.
 • Based on our results, primary care needs support from healthcare organizations to build capacity about CPPs and how to use them.

## Introduction

In primary care, patients’ reasons for care seeking are diverse and problems varies. General practitioners (GPs) face daily an enormous amount of ubiquitous symptoms which make it very complex to identify patients with potential cancer [1–3]. One out of ten patients that GPs encounter presents symptoms potentially indicating cancer, even so, cancer is seldom diagnosed by GPs in primary care [4,5].

GPs are often patients’ first healthcare contact when seeking care for symptoms potentially indicating cancer, hence they are important pieces of the puzzle in patients’ cancer caretrajectory [6]. Moreover, GPs have the potential to early identify patients with possible cancer when they interpret, assess and manage symptoms [7,8].

Multiple factors exist that may influence GPs’ decision to act upon a patient’s symptom presentation, such as different guidelines, pressure to reduce referrals, their own knowledge and skills [9] and time restraints [10]. Furthermore, some researchers suggest that GPs are more likely to suspect cancer in patients who seldom seek primary care [1,11], but also that increased consultation frequency in primary care is a risk marker for cancer [12–14]. Additionally, research suggest that GPs use their gut-feeling when suspecting cancer and making decisions about referral to secondary care [15,16].

Other studies indicate that the interaction between patient and GP is vital for cancer suspicion to arise during the encounter [1] and influences patients’ access to further care [17]. What seems to make it even more challenging is that patients and GPs have different perspectives about what counts as symptoms [8].

Alarms symptoms are increasingly being used as a way to enhance early diagnosis of cancer and determine referral from primary care to secondary care. This is the logic of ‘fast-track routes’, like standardized cancer patient pathways (CPPs) that are nowadays common in many countries: CPPs aim to ensure timely diagnosis for patients presenting with well-defined symptoms, so called alarm-symptoms [18]. In Sweden during 2015, CPPs were implemented as organizational tools for promoting early cancer diagnosis. The objectives with CPPs are to reduce waiting times, reduce regional difference, and increase patient satisfaction with cancer care, by standardizing the diagnostic process by regulating time frames for specified diagnostic procedures. CPPs intend to shorten the time interval between well-founded suspicion of cancer (presence of alarm symptoms and/or signs of suspected malignancy) and start of treatment [19], and to shorten the diagnostic work up for GPs, and thereby increase patient satisfaction. The problem that CPPs mainly aim to overcome is the unnecessary prolonged time interval before necessary investigations are performed that either confirm or reject cancer suspicion [19]. The implementation of CPPs might result in that GPs will think of cancer as a possible diagnosis in consultation in which they otherwise would not have done so since these referral guidelines are based on alarm symptoms to be attentive to [19,20]. Meaning that CPPs, nationally [19] and internationally [21,22] intend to guide GPs in identification of patients with symptoms (alarm symptoms), consequently ease GPs’ decision about referral to secondary care.

However, alarm symptoms of cancer, such as blood in stools, breast lumps, blood in urine and coughing in more than six weeks are common in the general population. Interestingly, even though alarm symptoms of cancer are common in primary care, they are seldom connected to cancer diseases [5,23]. A Danish study reports that about 15 percent of the population have experienced at least one of these alarm symptoms during the previous year [24]. Alarm symptoms thus have low positive predictive values (PPV) of cancer, indicating that the risk of having cancer when having a single alarm symptom is rather weak. However, combination of several alarm symptoms increase the PPV [25,26]. Thus, the PPV of a single symptoms is low, indicating that GPs require supplementary information to be able to decide whether to refer a patient or not [5,27]. Furthermore, patients with symptoms which are interpreted as non-alarming are less likely to be referred with CPPs and symptoms interpreted as vague are likely to result in a longer diagnostic interval [18], despite multiple contacts with the healthcare system [28,29]. Adding to the complexity, the fact that only half of all patients with cancer presents with alarm symptoms, according to GPs interpretation, makes the identification of these patients even more challenging [5,18,30].

Based on these outlined challenges, it seems that interaction in the encounter is a key phenomenon for how GPs handle the presented symptoms. According to symbolic interactionism (SI) human beings engage in actions to construct self, situations and societies [31]. Acting, interacting and interpreting are three fundamental premises of SI. *Action* entail that human beings act towards things, i.e. patients’ symptoms, based on their meanings of those things. *Interaction* entail that the meaning of those things or objects is derived from interactions, i.e. GPs’ encounters with patients in primary care. Lastly, these meanings are then managed and altered though an *interpretative process* by the person experiencing them, i.e. communication and reflection [32]. Similarly, the theory of SI can help us to extend our understanding about the complexity of how GPs handle these situations. This paper aims to explore how GPs assign meanings and act upon patients’ symptoms in primary care encounters in the context of CPPs.

## Material and methods

### Design, context, and participants

This study is based on individual interviews with GPs, who before participating in this study, also participated in a participant observational study in primary care [17]. All GPs who were observed (by the first and last author) in their daily practice when meeting patients with possible signs of cancer were invited to participate in individual interviews, all accepted to participate. The median time between the observation and the interview was four days, ranging between the same day up to 45 days post observation. Interviews were conducted with GPs working at four different primary healthcare centers, located in both urban and rural areas in one county in northern Sweden. The GPs’ work experiences ranged between less than one year to 27 years (median four years).

### Data collection

Data consist of material from thirteen semi-structured interviews with GPs, conducted by the first author (*n* = 12) and last author (*n* = 1). The interviews followed an interview guide with open-ended questions, also additional questions were asked based on emergent leads as well as questions that emerged from the previously conducted observations [17]. Questions about the GPs’ overall perceptions about encounters with patients seeking care for possible signs of cancer were the main focus, but questions regarding the previously observed encounter were also raised. Examples of question asked are ‘Can you explain how it is to meet patients with symptoms that might indicate cancer?’, ‘Can you describe how it is to listen to patients’ symptom presentation?’ and ‘Can you explain how it is to work with CPPs?’ All interviews were conducted during working hours at the GPs’ office at a day and time chosen by the GPs. Only the GP and the interviewer were present during the interviews. The interviews lasted between 31 and 48 min (median 39), were audio recorded and verbatim transcribed. Transcripts were then imputed to the software program MAXQDA version 2018 for coding, managing and analysis.

### Analysis

Data were analyzed following Grounded theory method (GTM) [31]. The process of coding started with initial coding, performed by the first author (CH) with continuous discussion with SH, ABC and ML. Secondly, CH performed focused coding, with support from SH, ABC and ML, whereby initial codes were sorted and grouped into clusters. Thereafter, working with these clusters, we continued with theoretical coding. Theoretical coding was performed to create categories, specify the relationships between the categories and even develop sub-categories within them. An overview of examples of codes, sub-categories and categories are presented in Table 1. Our analysis resulted in the model presented and discussed below. Theoretical coding was performed by all four authors.

**Table 1.** Overview of categories and examples of codes.

Examples of codes	Sub-categories	Categories	Core category
Diagnoses are dependent on presentations, Clear presentations makes it easy, Being guided by presentations, Needing details	Being dependent on patients’ stories	Sifting through patients' stories	Disentangling patients' care trajectory
Considering one minute as enough, Being frustrated when time is running, Being frustrated by bad presentations	Being frustrated by long and irrelevant stories		
Taking time to listen, Trying to sit back, Not being too eager, Not taking over with questions, Inviting patients to conversations	Allowing patients to tell their stories without interfering	Making sense of patients' symptoms	
Restricting presentations, Steering the conversation, Digging after wanted information, Matching symptoms, Looking for expected symptoms	Directing the dialogue and matching symptoms		
Feeling confident with CPPs, Not having to think, Makes it simple, Creates clarity, Creates structure	Leaning on CPPs for support	Feeling torn about CPPs	
Having more options without CPPs, Clinical assessment less valued, Feeling forced, CPPs as an additional task, Referring just because	Being frustrated by CPPs		

### Ethical considerations

Written informed consent was obtained from all participants and ethical approval was granted from the regional ethical review board (Dnr. 2017-296-31 M/2018-242-32M). Participation was on voluntary basis, meaning that GPs could withdraw from participation at any time.

## Results

**Figure 1.**
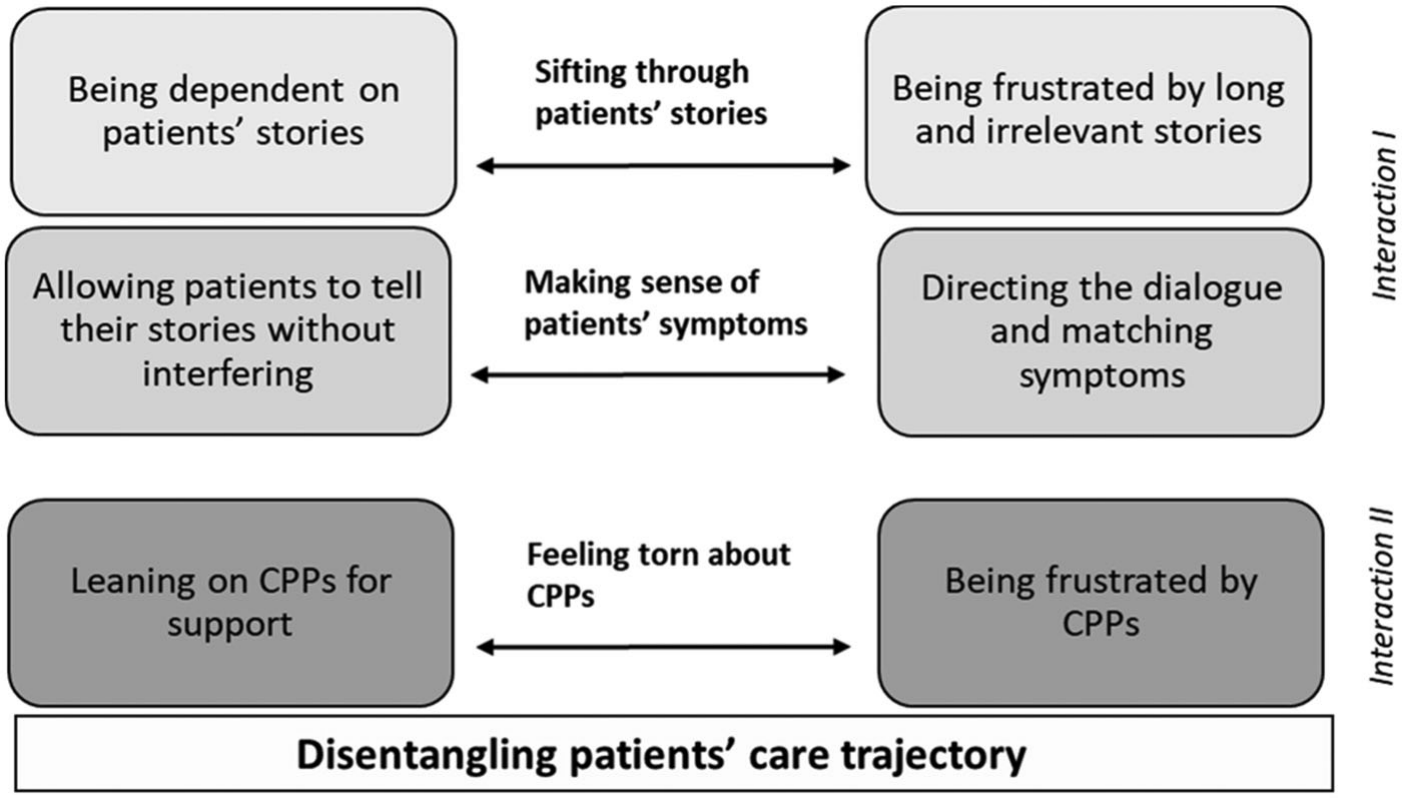
Conceptual model.

From our analysis, we developed a conceptual model (see Figure 1) that demonstrated how GPs assign meanings and act upon patients’ symptoms in order to determine next steps in patients’ care trajectory. Our core category *Disentangling patients’ care trajectory* depicts how GPs deliberate about conflicting and paradoxical aspects of the encounter, illuminating that patients’ trajectories are not as straight forward for GPs when using CPPs as these were intended. Paradoxical aspects stem in part from GPs facing two simultaneous interactions, one with patients’ symptoms in the encounter – and the other with CPPs within the healthcare organization. In deliberating about conflicting and paradoxical aspects of the encounter, GPs adopt different strategies depicted by three categories (see the center of the model). The first category *Sifting through patients’ stories* depicts the strategy of listening to patients’ presentations of symptoms, i.e. patients’ reasons for seeking care. The second category *Making sense of patients’ symptoms* depicts the strategy of interpreting these presented symptoms. The third category *Feeling torn about CPPs* depicts the strategy of assessing symptoms and reasoning about utilization of CPPs. Each of the three categories consists of two sub-categories with opposing directions (see arrows and boxes), thereby capturing tensions within each of the strategies developed by GPs. Our analysis shows that despite that standardized routines, such as CPPs, aim to aid GPs work in determining patients’ care trajectories, GPs face unintended aspects during the encounter that are conflicting and paradoxical, and that they must deliberate about.

### Sifting through patients’ stories

This category depicted the strategy GPs used for listening to patients’ presentations of symptoms, i.e. reasons for seeking care. One the one hand, GPs searched for a structured, precise and detailed presentation from patients. On the other hand, they filter through when patients talked too much and about irrelevant aspects. This category consisted of two opposing sub-categories.

The first sub-category depicted how GPs’ strategies depended on patients’ stories. GPs expressed that not merely what patients say is crucial, also how patients tell their stories is important for GPs to be able to interpret patients’ health status and to legitimize the presented symptoms. GPs described that they want patients to give a quick, structured and coherent presentation of their problems. Patients’ presentations were described as a very important part of the encounter, having much at stake. One GP expressed that diagnosis are 80–90% dependent on the patient, which illuminates that patients have a crucial and difficult task when presenting their symptoms to GPs in primary care. Patients’ presentations were also considered to be indicative for further steps of investigation and treatment.

GPs described a need of getting answers to certain questions, questions regarding patients’ specific symptoms. Also, GPs described that they need information from patients in terms of duration of symptom, time course, how the symptoms have changed, and if there are any other associated symptoms. GPs described that they want patients to deliver a detailed chronological explanation about their experienced sensations. Challengingly, GPs described that they wished for information that was not available and expressed that they wished that patients would have a better memory for details, since they considered patients many times give poor description of their symptoms.

I want to know, well, know exactly how it has been and during what time, how it has changed, and answers to specific B-symptoms, and weight loss to be able to form myself some kind of opinion about how likely it is that it is cancer (Interviewee 7)

The second sub-category depicted how GPs’ were frustrated by long and irrelevant stories. GPs expressed that even though they were dependent on patients’ stories, they felt irritated when patients talked ‘too much’ since it was described as very challenging to listen to desultory and long presentations/stories, which is a contradiction from the first sub-category. According to GPs, many patients fail with presenting their reasons for care seeking, since it was described that patients tend to present in an unclear manner, lacking structure and that presentations often floated away from the topic. GPs described being impatient and that they thought ‘get to the point’ while listening to patients’ presentations. It was described as frustrating when time passes during, what GPs perceived, as bad (e.g. long, unstructured, unspecific) presentations, and since time is limited during encounters these feelings became even more influential. Hence, stress during working hours seemed to influence GPs frustration while listening to patients’ presentations.

One minute is enough for patients to tell what I need to hear to be able to continue with the investigation, it might be enough with one minute, but patients, they don’t know how to describe their symptoms, so they can be talking for like 15 minutes and it can be, it can be about all kinds of symptoms, relevant and irrelevant, it can be about stuff that happened 10 years ago… (Interviewee 3)

### Making sense of patients’ symptoms

This category depicted the strategy GPs used for interpreting patients’ presented symptoms. GPs attempted to balance between being passive, in terms of giving patients room to act, with being the active part, in terms of steering the conversation, and matching symptoms. This category consisted of two opposing sub-categories.

The first sub-category captured how GPs allowed patients to tell their stories without interfering. GPs described that listening is essential for grasping patients’ health complaints and concerns. The GPs invited patients to conversations, often by starting the encounter with an open-ended question to get the conversation going, allow patients to steer the first part of the presentation, and give room for patients to present their health complaints. However, GPs described that they need to prevent themselves from being too eager during the encounter, meaning that they actively have to try to sit back and listen without interfering and take over the conversations with questions. By listen to patients’ stories GPs were able to create a perception about the presented problems and patients’ worries. It was illuminated that GPs have to balance between being a passive listener and being an active questioner, which was described as challenging since GPs described the importance of taking time to listen to patients’ presentations, even though they have follow-up questions to ask.

We have learned, and I think that is a good idea, that you should start with asking open-ended questions so that patients are given the opportunity to give their side of the story, I think that is important, but at the same time you can be impatient and, eeh, wanting to ask follow-up questions and concretize based on the suspicion you have… (Interviewee 11)

The second sub-category depicted how the GPs directed the dialogue and matched symptoms. GPs expressed that they felt a need to steer patients’ in their presentations, since they experienced that it is not optimal to allow patients to speak freely, which was illuminated by GPs actions in those situations when they perceived that patients gave insufficient and unspecific presentations. GPs described that they need to interfere and steer patients in their presentation to keep them on what they perceived as the right track. Also, GPs described using closed-ending questions to be able to pilot patients towards those aspects which GPs consider as most essential, and that they asked specific questions if patients did not present what they expected – the ‘right’ information.

Even though patients’ presentations were described as important and as essential parts of the encounter, some GPs emphasized that they are driven by ‘a feeling’ they get when listening to presentations. It was described that this feeling is based on what and how patients present and that this ‘feeling’ functions as a point of departure when sifting through symptoms and matching symptoms, aiming for identification of which symptom to lay focus on. The GPs expressed this as a strategy for evaluating and assessing symptoms, as well for making decisions about following acts. GPs described that if they get a suspicion about something malignant, they match patients’ presented symptoms with, for example, criteria for CPP. Also, GPs described that they match presented symptoms in terms of interpreting whether the patients fit into the frame, hence, having symptoms that fulfil criteria for standardized routines such as CPPs, or not.

Furthermore, GPs expressed that they felt like a detective when trying to screen for possible malignancy and to understand how the symptoms may link together, especially since the picture they get often roils by all different health complaints patients express during encounters. The perceived need of screening, matching symptoms, and acting like a detective was described as frustrating, especially during stressful hours, our when patients’ presentations were perceived as insufficient and unspecific.

It is like a big ocean of symptoms, well many experiencing a variety of symptoms and most of them are not malignant, but there are some you are supposed to find, and that is the challenge of course (Interviewee 4)

### Feeling torn about CPPs

This category depicted GPs strategy for assessing symptoms and reasoning about utilization of CPPs. On the one hand GPs considered CPPs as valuable tools in their daily practice. On the other hand, GPs perceived being limited by CPPs and that CPPs create higher demands in primary care. Again, this category consisted of two opposing sub-categories.

The first sub-category captured that following CPPs is like reading a manual, covering that CPPs ease decisions for referral by identifying and legitimizing alarm symptoms, hence CPPs provide GPs with support. GPs described how alarm symptom ‘starts something within’ GPs, that alarm symptoms make GPs immediately react. Alarm symptoms were described as being ‘programmed’ into GPs head during education which make them react and act, since these ‘red flag symptoms’ are imprinted and embedded in their minds, hence being an essential part of GPs expertise. Furthermore, GPs described that they become more meticulous and increase their vigilance when alarm symptoms are present, and that they value alarm symptoms above their gut feeling. Additionally, alarm symptoms were described to have an important and indicative function which help GPs facilitate identification of the problem and deciding about further referrals such as CPPs.

Furthermore, CPPs were described to support GPs in their decisions, since CPPs clarifies, facilitates, and provides structure and guidelines to follow. Hence, GPs described that they did not need to find a new solution by themselves anymore, they just had to follow what was written in the instructions for CPPs. GPs explained that these routines made them feel confident in their role as medical expertise and that CPPs increase the credibility of their work. Also, CPPs were described to make GPs work easy, one GP compared CPPs to a cookbook and said that it is just to read step by step and do what it says.

For me in primary care it is easy to fall back on these [CPPs], like okay yes criteria are fulfilled, lets send a referral (Interviewee 5)

The second sub-category captured how GPs experienced being tied by rules and unable to work freely as clinician due to CPPs. It was described that CPPs sometimes create frustration for several reasons. Firstly, GPs described that they feel constrained by CPP, due to feeling forced to follow CPPs, and feeling that CPPs take away opportunities/choices about what needs to be done in terms of further investigations. Secondly, even though GPs expressed that guidelines are not everything that matters, they perceived that CPPs have got more power than clinical assessments, meaning that GPs considered their clinical assessment have been devalued after the implementation of CPPs. Thirdly, GPs described that CPPs create higher workload without more resources since more tasks are supposed to be done by primary care, which was expressed as frustrating.

Sometimes I feel that we refer more patients because we feel constrained by CPPs, well just because it should be done … now we feel sometimes somewhat tied by rules so we send a referral to, for example colonoscopy, which we might not have done five years ago (Interviewee 1)

Furthermore, our analysis showed that GPs have differences in knowledge and information about CPPs. All GPs described having heard about CPPs, but their extent of information about these standardized routines varied, some GPs expressed a perceived lack of information about CPPs and that they had underused CPPs because of that. Thus, those GPs who expressed having limited information about CPPs faced feelings of uncertainty and confusion regarding how to use CPPs, how to write referrals and how to get access to already existing information about CPPs, which following extract illuminates.

I am a bit bad at this with CPPs, when to use them, I must say, I guess I have underused them… Well, I am a bit uncertain about when I can add CPP (Interviewee 9)

## Discussion

Our results illuminated three strategies GPs used to assign meanings and act upon patients’ symptoms in the encounter with patients. These results can be best interpreted through the lens of symbolic interactionism because it enables us to understand how GPs interpret symptoms through the symbols used to communicate (namely oral and body language), and how these meanings influence their actions and how the process of interpretation guides decision making and actions regarding the next steps in patients’ care trajectory. According to SI human beings are social beings who are inseparable from the society, both being created by social interactions and understood by the meaning of the other [33]. As explained previously, SI encompasses three premises.

Firstly, human beings act toward things on the basis of the meanings that things have for them [32]. That is, a person does not respond directly to the situation per see, but rather based on what meaning the situation has to the person [33]. In relation to our results, that means that GPs act, e.g. make decisions regarding patients’ care trajectory, based on the meanings they assign to patients’ symptoms. As our first strategy, *Sifting through symptoms,* illuminates, GPs are not responding directly to the symptoms as objective things but rather to patients’ symptoms as communicated symbolically using language including body language. Our results depicted how GPs are dependent on patients’ stories but also frustrated when these were long and winding, which sheds light on the underlying challenges of the encounter. Adding to the complexity, previous research finds that patients’ initial presentation have a predictive value itself [34], and that it is possible to reduce cancer diagnostic delays by asking cancer related questions during the initial phase of the encounter [35]. These findings, in combination with ours, illuminate the complexity of assigning meanings to patients’ symptoms, and highlights the importance of the initial phase of the encounter.

Secondly, the meaning of things is derived from social interactions [32]. In other words, one person’s action create meaning for the other person [33]. As our second strategy, *Making sense of patients’ stories* demonstrates, GPs assign meaning to patients’ symptoms in the process of interaction with patients who themselves act to define their symptoms. GPs get access to patients’ stories and symptoms through interacting with them, hence, interacting with patients is indeed crucial for assigning meaning to presented symptoms. Our results depicted how GPs interact with patients in term of inviting patients to dialogue, but also by steering the conversation. These findings illuminate that social interaction is essential in a healthcare seeking context. Furthermore, as our preceding study shows, GPs and patients are negotiating symptoms during the encounter in an interactive process [17]. Besides, the importance of interaction during encounters is found elsewhere, highlighting GPs experiences of having to steer the dialogue to be able to assign meaning to symptoms [36]. These findings illuminate that social interaction is crucial, yet complex, in a healthcare seeking context, which is also understood in this study.

Thirdly, meanings are handled and altered in interpretative processes [32]. In our study, GPs assign meaning to patients’ symptoms through an interpretive process that is constantly changing and open to redefinition. As our third strategy illuminates, *Feeling torn about CPPs*, GPs attempt to align their knowledge of alarm symptoms from CPPs with the meanings they assign to patients’ symptoms in the encounter. In this sense, despite the attempts of CPPs to offer fixed guidelines, meanings are emerging during the encounter. Our results depicted how GPs follow CPPs like an instruction manual but also feel constrained by these rules. They thereby assign meaning to patients’ symptoms through a simultaneous and parallel interaction with CPPs in the healthcare organization, something that patients are not necessarily interacting with. GPs’ knowledge about cancer alarm symptoms is nothing new, it is a big part of their medical expertise, with its’ importance highlighted in previous research [1,6]. Furthermore, one study has found that GPs readiness to act on symptoms is associated with improved cancer survival, and that Swedish GPs are more prone to act on symptoms than for example GPs in UK [37]. Nevertheless, GPs sometimes fail to recognize specific alarm symptoms [38], indicating that GPs do not respond directly to symptoms as objective things and that GPs assign meanings to symptoms by interacting with patients. Besides, since the introduction of CPPs the playing field has changed, meaning that GPs now have to match their knowledge through interacting with CPPs’, not only with patients.

Thus, our model captures these two simultaneous interactions situations – one with patients’ symptoms in the encounter and the other with CPPs within the healthcare organization – that produce a broader contradiction for GPs in terms of how to assign meaning to patients’ symptoms. On the one hand, GPs adopt strategies according to their interaction with CPPs, thus CPPs are shaping GPs interpretations of patients’ symptoms. On the other hand, GPs adopt strategies according to their interaction with patients which means that even with the implementation of CPPs, CPPs are always being re-created through GPs own interpretations of patients’ symptoms. A recent study from Denmark reports that even though nine out of ten patients with ovarian cancer presented symptoms in primary care prior to diagnosis, only 36% of these patients were referred with CPP, indicating that CPPs are not enough to ensure early detection of cancer [39]. It is reasonable to discuss whether GPs could benefit from additional support to CPPs when facing patients with symptoms indicating cancer. Green *et al.* suggest that a supplementary option to CPPs could be beneficial, allowing patients to be urgently referred to secondary care in cases when cancer is not suspected, and when patients do not fit into the frame for CPPs [6]. Furthermore, a recent study from Sweden finds that healthcare professionals need improved information about CPPs [40], which also some GPs in our study expressed. These findings from previous research, in combination with ours, indicate that the implementation of CPPs, at least in the studied region, in primary care still needs to be supported and improved to be able to function optimally well. Although the CPPs are implemented in Sweden since a time ago and big efforts have been done to facilitate the implementation there are still various utilization challenges in different regions in Sweden. Consequently, CPPs need to be refined and information improved. Besides, a vigilant discussion about the utility, shortcomings and ways forward is called for.

### Strengths and weaknesses of the study

One major strength with this study is that it is succeeding from an observational study. Meaning that all participating GPs have had at least one encounter observed by the first and/or last author. Hence, the researchers have had greater insights about encounters with patients and could develop interview questions accordingly. Also, all GPs who had their encounter observed [17] participated in this study which we consider strengthening. Furthermore, the authors’ have diverse backgrounds (public health, sociology, medicine and nursing) which has been highly complementary to each other and beneficial for the development and result of this study. Lastly, using SI strengthens our study, since it further enables our results to be transferable to other settings and contexts. Furthermore, SI is a theory developed for examining micro level processes, making it highly suitable for exploring GPs’ perspectives of the encounter with patients. Also, SI is concerned with but goes beyond the spoken language and communication to capture actions, which we consider highly relevant in this study.

However, our study has some weaknesses. Firstly, we have a rather limited representation from different primary healthcare centers, despite that, we have a diversity in size and location. Primary healthcare centers located in both urban and rural areas participated. Secondly, when conducting interview studies there is always a risk for recall bias [41], however, we were not mainly interested in a particular encounter, instead we asked general question about encounters with patient who sought care for possible cancer symptoms. Hence, we assume that the risk of recall bias was decreased. Thirdly, the process of interviewing until saturation, emphasized by Charmaz [31], was not applied since the sample in this study consist of the total sample of GPs participating in the observational study [17]. However, we assess that theoretical categories constructed in this study are saturated and grounded in the data.

### Conclusion and implications for policy and practice

Standardized cancer patient pathways is one of the biggest reforms in the history of Swedish healthcare, primarily aiming to optimize patients’ diagnostic pathways with quality and timeliness. It is also aiming to support and ease GPs’ work of deliberating how to manage patients’ symptoms and complaints when suspecting cancer. However, to carry out patient/person-centered dialogues, interpret presented symptoms and link to CPPs is a big and essential challenge during the encounter. Also, there is no such thing as a ‘standardized patient’, so providing standardized care might challenge the provision of patient/person-centered approach and taking the whole person into account.

If standardized routines, like CPPs, are the future within healthcare, it is essential that GPs are given opportunities at their workplaces to continuously be informed and be supported in order to practice CPPs and thereby optimize trajectories for patients undergoing cancer diagnostics. This implicates that stake holders and the healthcare organization need to continuously work with improvements of infrastructure about CPPs and ensure that GPs have access to support and information that facilitates their daily practice. Additionally, it is also necessary that the channels from which they can access information from are easy accessible, updated, and well known by GPs.
